# Impact of Low-Dose Dronabinol Therapy on Cognitive Function in Cancer Patients Receiving Palliative Care: A Case-Series Intervention Study

**DOI:** 10.1089/pmr.2023.0024

**Published:** 2023-12-07

**Authors:** Ditte Buchwald, Casper Schmidt, Dorte Buchwald, Kristina Iris Winter, Ivan Bo Nielsen, Kirsten Klostergaard, Dorte Melgaard, Steen K. Fagerberg, Peter Derek Christian Leutscher

**Affiliations:** ^1^ Centre for Clinical Research, North Denmark Regional Hospital, Hjørring, Denmark.; ^2^ Department of Communication and Psychology, Aalborg University, Aalborg, Denmark.; ^3^ Palliative Care Team, Department of Gerontology, North Denmark Regional Hospital, Hjørring, Denmark.; ^4^ Department of Clinical Medicine, Aalborg University, Aalborg, Denmark.

**Keywords:** cancer, cognitive impairment, medical cannabis, pain management, sleep disturbances

## Abstract

**Background:**

Cannabis may offer therapeutic benefits to patients with advanced cancer not responding adequately to conventional palliative treatment. However, tolerability is a major concern. Cognitive function is a potential adverse reaction to tetrahydrocannabinol containing regimens. The aim of this study was to test cognitive function in patients being prescribed dronabinol as an adjuvant palliative therapy.

**Methods:**

Adult patients with advanced cancer and severe related pain refractory to conventional palliative treatment were included in this case-series study. Patients were examined at baseline in conjunction with initiation of dronabinol therapy and at a two-week follow-up using three selected Wechsler's adult intelligence scale III neurocognitive tests: Processing Speed Index (PSI), Perceptual Organization Index (POI), and Working Memory Index (WMI). Patients were also assessed using pain visual analog scale, Major Depression Inventory, and Brief Fatigue Inventory.

**Results:**

Eight patients consented to take part in the study. Two patients discontinued dronabinol therapy, one due to a complaint of dizziness and another critical progression of cancer disease, respectively. The remaining six patients were successfully treated with a daily dosage of 12.5 mg dronabinol (*p* = 0.039). PSI (*p* = 0.020), POI (*p* = 0.034.), and WMI (*p* = 0.039).

**Conclusions:**

Cognitive function improved in this group of patients with advanced cancer in conjunction with low-dose dronabinol therapy. The cause is likely multifactorial including reported relief of cancer-associated symptoms. Further clinical investigation is required.

## Introduction

Cancer is the second leading cause of death globally, and it was responsible for an estimated 9.6 million deaths in 2018.^1^ Treatment for some cancers has become more effective in recent years, with longer and increased survival.^2^ However, disease-related symptoms, such as pain, poor appetite, sleeping disturbances, and depression, can cause poor quality of life (QoL).^2–4^ Pain affects the majority of patients with advanced cancer.^5^ Opioid-based pharmacotherapy is a main strategy in conventional management of pain in cancer patients. However, opioids do not always provide adequate pain relief, and they are also associated with bothersome side effects.^2,3^

When conventional medicine does not relieve the pain adequately, other treatment options are sought. For that reason, cannabis products have attracted increasing attention among cancer patients receiving palliative care in the past decade, despite their limited clinical evidence regarding their effects and safety of cannabis, which also makes physicians reluctant to prescribe them.^6–8^

However, larger empirical datasets with solid clinical grounding are currently being collected. This will gradually garner clinical evidence addressing cannabis products used in palliative care. As a result, cannabis is viewed today as a potential complementary therapeutic approach along with conventional palliative regimens, particular for the treatment of pain, nausea, vomiting, and other cancer-related symptoms.^9,10^

Studies evaluating recreational cannabis users have suggested a concerning increased risk for developing dyscognitive manifestations, likely related to tetrahydrocannabinol (THC).^11,12^ Some studies suggest that THC induces lasting cognitive impairment across multiple cognitive domains. These especially include working memory, attention, and executive functions such as planning, reasoning, interference control, and problem-solving.^11,13–19^

Cancer, its treatments, and supportive medications can each cause cognitive impairment and its related sequelae.^20–22^ Cognitive deficits negatively affect QoL. They can also lead to distorted well-being, stress, depression, and anxiety.^23–25^

It is, therefore, important to explore the potential impact of cannabis on cognition among cancer patients receiving palliative care.^26^ Few studies have investigated cognition among this population, and none have revealed a deteriorating effect of cannabis on cognitive functioning. However, the studies are characterized by different patient groups, cannabis products, and administration routes in combination.^12,27,28^

This study aimed to assess cognitive changes in a subgroup of Danish patients with advanced cancer who are scheduled for initiation with a standardized dronabinol regimen as adjuvant pain-relieving therapy in conjunction with conventional palliative care.

## Materials and Methods

This case-series study was conducted in the palliative care outpatient clinic at the North Denmark Regional Hospital from January to April 2020. The interdisciplinary palliative care team conducts patient visits primarily in the patient's home or, alternatively, in the hospital. On an annual basis, 700 patients with advanced disease are referred to the team.

### Participants

Study inclusion criteria included age ≥18 years, active advanced cancer disease, planned dronabinol therapy to manage pain refractory to conventional palliative interventions, no prior cannabis-related therapies used, and ability to comprehend an informed written consent form. Those with cerebral metastasis, severe mental disorder, or dementia were excluded from participation.

### Treatment

The patients received treatment with dronabinol as an oral oil solution (25 mg/mL) in accordance with existing guidelines in the palliative care outpatient clinic. A prescription for dronabinol, as a magistral product (30 mL bottle), was issued to each patient by the palliative care physician, to be obtained from the local pharmacy. All patients were treated with 2.5 mg, equal to three drops of 0.83 mg each for the initial three days. The dosage was subsequently increased in accordance with a standard titration plan (Table 1).

**Table 1.** Plan for Scheduled Titration of Daily Dronabinol Dosing

Day	Drops (***n***)	Dosage (mg)	Daily frequency	Total daily dosage (mg)^a^
1–3	3	2.5	Once a day	2.5
4–6	3	2.5	BID	5.0
7–9	3	2.5	TID	7.5
10–12	4	3.3	TID	10.0
13–15	5	4.2	TID	12.5

BID: Twice a day; TID: Three times a day.

^a^ Rounding of total daily dosage.

BID, bis in die; TID, ter in die.

The patients were instructed to follow the scheduled titration plan with referral to effects and side effects experienced by the patient. Hence, the daily dosage was increased if pain persisted, with a maximum intended total daily dosage of 15 mg. The patients were also instructed to discontinue therapy and contact the palliative team if any unacceptable side effects occurred, such as nausea, vomiting, dizziness, or somnolence.

### Study design

Informed consent was obtained from eligible patients at their visit scheduled for dronabinol initiation. The study consisted of three study sessions: (1) Pretest session, (2) Test session I (*baseline*), and (3) Test session II (*Two-week follow-up*). Each session was conducted in the patient's own residence or, alternatively, the hospital or hospice if a patient was admitted during the study period.

The first test session (*baseline*) was held one to four days before dronabinol initiation. All tests were conducted in the morning. Each patient completed a brief unstructured interview to gain the patient's subjective experience about the treatment and to evaluate the patient's ability to participate in the study, in addition to neuropsychological testing conducted by a psychologist and a physical examination.

After two weeks of treatment with dronabinol (*follow-up*), the second test session was performed at day 14 or 15, also in the morning. The patients underwent the same assessments as at baseline. This session was initiated approximately one hour after the morning dose of dronabinol oil drops. The timeline was chosen according to pharmacokinetic studies showing the median peak time is achieved after 45 to 60 minutes and median plasma concentration of metabolite 11-OH-THC peaks after 60 to 90 minutes.^29^

### Neuropsychological testing

As part of the neuropsychological testing performed by the same clinical psychologist, each patient completed three different cognition subtests from the Wechsler's adult intelligence scale III (2007) before initiation of dronabinol (baseline) and two weeks later (follow-up): Working Memory Index comprising the Digit Span (forward and backward) subtest measuring short-term memory and working memory, Processing Speed Index measuring the speed of mental processing, and Perceptual Organization Index measuring nonverbal and in-the-moment reasoning.

Furthermore, to determine whether patients experienced any change in clinical symptoms or health-related measures, which could potentially impact cognitive performance, each participant completed a battery of clinical state assessments. These included the visual analog scale (VAS) measured pain. The Brief Fatigue Inventory (BFI) measured the severity and impact of cancer-related fatigue. The Major Depression Inventory (MDI) measured of depression at baseline and at follow-up. In addition, any changes in the patient's use of conventional medication were registered at the follow-up session.

### Ethical approval and consent to participate

Participation in the study required an informed written consent to be signed by the patient. The Regional Ethics Committee in Region North Denmark was contacted and waived the need for their ethical approval of the study (reference no. 2019-000199-116) as the study was defined as a clinical quality assurance study investigating the risk of cognitive adverse outcome in conjunction with already planned dronabinol therapy as complementary palliative care.

### Statistical analyses

Descriptive data were registered at baseline and at follow-up. Because of the small related sample and as the assumption of normality is questionable, a Wilcoxon test was conducted to assess in-group cognitive changes from baseline to two-week follow-up in each of the three cognitive domains. A two-tailed *p*-value of 0.05 or less was considered statistically significant of these limited available data for this population. A *z* score was used to determine the standard deviation from the mean. Statistics were calculated in IBM SPSS Statistics 24.

## Results

Eight patients planned for dronabinol therapy in the palliative care outpatient clinic were invited to participate in the study, and they all consented to participate. However, two patients were not able to complete the study. One patient stopped treatment with dronabinol after four days due to side effects (dizziness). Since we could not test whether dronabinol affected cognition at the follow-up test, the patient was excluded from the follow-up analysis. The second patient's cancer progressed to the extent that continuation in the study was not possible. In total, six patients completed the quality assurance study, and all six patients were titrated to a total daily dosage of 12.5 mg dronabinol.

Patient demographic and clinical characteristics are presented in Table 2. The median age of the patients was 75 years, ranging from 54 to 80 years. Five patients were female, and one was male. All patients had earned at least a high school diploma. None of the patients were receiving chemo- or radiation therapy.

**Table 2.** Patient Demographic and Clinical Characteristics

Patient	Sex/age (years)	Educational level	Diagnosis	Oncology therapy	
				Prior	Current
1	F/57	High school	Malignant melanoma	Chemo	None
2	F/75	College	Breast cancer	Radiation	None
3	F/74	University	Pancreas cancer	Chemo	None
4	F/54	College	Parameningeal sarcoma	Chemo	None
5	F/80	College	Malignant melanoma	None	None
6	M/80	University	Prostate cancer	Chemo	None

F, female; M, male.

### Patient-reported treatment outcomes

At the follow-up visit, all six patients reported in the interview an adequate pain-relieving effect from the dronabinol treatment based on a titrated daily dosage of 12.5 mg. Hence, no patients needed further titration beyond this dosage. Moreover, the patients also reported other beneficial treatment outcomes, including reduction or discontinuation of conventional pain-relieving medication with a corresponding decrease in side effects (e.g., improved opioid-associated dizziness, less fatigue, increased mobility, and QoL). Visual hallucination (as a single episode) was reported as the only major noticeable side effect (Table 3).

**Table 3.** Patient Finally Dronabinol Titrated Dosage Level and Reported Treatment Outcomes at Two-Week Follow-Up

Patient	Final dronabinol titration level (mg/day)	Pain	Side effects	Other reported treatment outcome
1	12.5	Reduced	None	Discontinued/reduced conventional medication Improved life quality
2	12.5	Reduced	None	Discontinued/reduced conventional medication Less fatigue
3	12.5	Reduced	None	Do no longer sleep during the day Able to walk with dog
4	12.5	Reduced	Visual hallucination (single episode)	No longer bedridden
5	12.5	Reduced	None	
6	12.5	Reduced	None	Discontinued/reduced conventional medication

### Changes in cognitive performance

Cognition was improved at follow-up in each of the three tested domains (Table 4). This was true for speed and mental processing, with a significant increase in the median value of 7 at baseline to 9 at follow-up (*Z* = 2.33, *p* = 0.020). Likewise, in-the-moment reasoning also improved significantly from 6 at baseline to 10 at follow-up (*Z* = 2.12, *p* = 0.034). Finally, short-term and working memory also improved significantly from 5 at baseline to 7 at follow-up (*Z* = 2.06, *p* = 0.039).

**Table 4.** Wechsler's Adult Intelligence Scale III Cognition Test Results at Baseline vs. Two-Week Follow-Up

WAIS III cognition test	Baseline (T0)	Follow-up (T2 weeks)	***p***
	Median	Median	
Short-term memory and working memory (WMI)	5	7	0.039
Speed of mental processing (PSI)	7	9	0.020
Nonverbal and in-the-moment reasoning (POI)	6	10	0.034

POI, Perceptual Organization Index; PSI, Processing Speed Index; T, therapeutic cannabis; WAIS, Wechsler's adult intelligence scale III; WMI, Working Memory Index.

### Patient rating of pain, fatigue, and depressive symptoms

In accordance with the pain relief reported by the patients during the collection of patient-reported outcomes at the follow-up visit, the median VAS (pain) score decreased significantly from 5 at baseline to 4 at follow-up, *Z* = 2.06, *p* = 0.039 (Table 5). Among the six patients, the VAS score declined by >30% in two patients (70% and 33%, respectively) and <30% in the remaining four patients (25%, 20%, 11%, and 0%, respectively). A similar declining tendency, also statistically significant, was observed for the patient rating of depressive symptoms with an MDI score of 9 at baseline versus 6 at follow-up, *Z* = 2.02, *p* = 0.043. Finally, fatigue was also improved significantly from a BFI score of 5 at baseline to 4 at follow-up, *Z* = 2.02, *p* = 0.043.

**Table 5.** Patient Rating of Pain, Fatigue, and Depressive Symptoms at Baseline vs. Two-Week Follow-Up

Patient rating	Baseline (T-0)	Follow-up (T-2 weeks)	***p***
	Median	Median	
Pain (VAS)	5	4	0.039
Fatigue (BFI)	5	4	0.043
Depressive symptoms (MDI)	9	6	0.043

BFI, Brief Fatigue Inventory; MDI, Major Depression Inventory; VAS, visual analog scale.

## Discussion

This case series aimed to evaluate how dronabinol, as adjuvant therapy to conventional palliative pain-relieving treatment, affects cognition in patients with advanced cancer. To our knowledge, the current case series is the first of its kind to investigate for potential changes in cognitive performance before and after dronabinol titration for pain relief.

The results of this study suggest that treating palliative cancer patients with dronabinol for 14 days does not impair cognition. Rather, it seems to improve cognition in different domains, including in speed of mental processing, nonverbal and in-the-moment reasoning, as well as short-term memory and working memory. Furthermore, results from this study suggest some improvements in multiple self-reported relevant measures of clinical state.

The patients reported a reduction in pain, depressive symptoms, and fatigue in conjunction with dronabinol treatment. All three symptom entities have a negative impact on cognition.^30–32^ One possible explanation for this effect is that reduction in pain, fatigue, and depressive symptoms in conjunction with dronabinol treatment may contribute to improved cognition.^31,33^ However, other variables, such as the cancer disease, comorbidity, and other medical treatment might also affect cognition.^22^

The improvement in the various clinical parameters is consistent with other recent reports, which also have demonstrated varying degrees of beneficial outcome for patient, treated with cannabis products.^34–37^ None of the patients included in the study had their pain medication doses changed by their palliative care clinicians during the two-week study period. However, three of the six patients reported in the second health interview that their pain was so well treated with dronabinol that they had stopped their treatment with conventional pain-relieving agents, such as opioids, from which they had experienced negative side effects, particularly tiredness and nausea.

Another study involving patients with cancer receiving treatment with cannabis products also suggested a subsequent reduction in opioid medication use.^38^ Importantly, a causative relationship between the implementation of dronabinol and a decline in opioid usage in relation to improved cognition cannot be determined with this study.

Our finding of improved cognition is in opposition to current theory and studies regarding THC among recreational users.^39–42^ However, our findings are supported by three other recent studies that found improved cognition and general health among patients using cannabis products.^12,27,43^ These studies included different patient groups (none were receiving palliative care) receiving cannabis by different routes of administration (smoked, inhaled, and oil), and treatment did not follow a titration regimen. Some studies have suggested that the route of administration and dosing titration of cannabis may have an influence on the risk of cognitive impairment.^13,16,44^

As opposed to the other studies, our palliative cancer patients were treated with dronabinol administered as an oil product. The daily dosage was also titrated carefully after the recommendation to “start low, go slow, and stay low.” This allows the body to adapt to the pharmacologically active molecules, for finding the therapeutic window for the relief of symptoms, and at the same time operate in a treatment scenario controlling the risk of side effects to occur. Consequently, the results from the other studies are not directly comparable with those in our study.

Longitudinal studies have shown that the initiation of cannabis consumption among teenagers and young adults seems to be important for impaired cognitive development.^45^ This effect is hypothesized to occur because cannabis is particularly more harmful to the developing brain in individuals younger than 25 years compared with the mature brain in individuals above that age.^41,46^ When investigating the effects of cannabis in humans, Wilson et al. found that the early onset of cannabis use is associated with a lower percentage of gray matter and a higher percentage of white matter compared with late-onset users.^47^

In our study, all patients were older than 50 years, and patients in this age group may theoretically not be as vulnerable to dronabinol's persisting negative cognitive impact as younger patients. It cannot be ruled out that the patients in our study have used cannabis for recreational purposes when younger, and, therefore, a final conclusion cannot be drawn. Few studies have tested the age hypothesis.^48,49^

Interestingly, recent studies have shown that treatment with THC restores cognitive function in aging mice; the increased level of Sirtuin1 (an enzyme that has been previously shown to be involved in neuroprotection and neuroplasticity) was elevated in the hippocampus and the frontal cortex of old mice.^50–52^ Age seems to be of major importance regarding whether there is an improvement or an impairment in cognition when using dronabinol.

Another factor of interest is the possible “placebo effect.” Many of the participants had struggled to get access to cannabis prescribed by a physician, and participation in this study achieved that goal. This situation may have created a placebo effect, which itself could, at minimum, contribute to the relief of symptoms.^53,54^ To understand the mechanism of placebo effects, there is not just one placebo effect, but multiple.^55^ From a psychological point of view, different mechanisms can contribute to the placebo effect.

These include expectations, conditioning, learning, motivation, somatic focus, reward, and reduction of anxiety.^53,54^ It is known that for many patients receiving palliative cancer treatment, their motivation for starting treatment with cannabis is based on the hope of surviving cancer, based on the rationale that cannabis may contain curative properties.^7^ Relief of symptoms is often perceived as a secondary reason for treatment. The patients often have high expectations for dronabinol to be beneficial.

Other limitations should also be considered. The small sample size limited the study's statistical power. Therefore, the dronabinol treatment results should be interpreted only as indicative of cognitive improvement. Enrolled patients were also given many different medicines. This study was underpowered to explore the statistical significance of pharmacological interactions. In addition without a control group, we were not able to establish causality between dronabinol treatment and improvement in the patients' well-being.

Lastly, as the study retested the patients with the same neuropsychological assessments, we cannot exclude the possibility of learning effects, which may have contributed to the outcome date. We have attempted to minimize this potential confounding effect by selecting tests that do not require finding the correct answer. Instead, the tests are designed for a clinical setting, and the purpose is to examine changes between the sessions.

## Conclusion

Six out of eight palliative cancer patients completed neuropsychological testing before initiation of dronabinol therapy and at two-week follow-up. It was found that in this group of patients, the short-term use of dronabinol did not impair cognition. Rather, the treatment was associated with improved cognition, especially in the processing and reasoning domains. The study also found relief of pain, fatigue, and depressive symptoms, which may have had an indirect beneficial effect on cognitive functions.

Moreover, some of the patients reported a decrease in conventional pharmaceuticals, notably opioids. The study results suggest dronabinol may have a beneficial effect on different parameters for patients with advanced cancer receiving palliative care. However, this study was small and nonrandomized, so placebo effect may be a cofounder. More research is warranted to explore this important area of cognitive outcome related to cannabis therapy among patients receiving palliative care.

## Abbreviations Used

BFI: Brief Fatigue Inventory
BID: bis in die
F: female
M: male
MDI: Major Depression Inventory
POI: Perceptual Organization Index
PSI: Processing Speed Index
QoL: quality of life
T: therapeutic cannabis
TID: ter in die
THC: tetrahydrocannabinol
VAS: visual analog scale
WAIS: Wechsler's adult intelligence scale
WMI: Working Memory Index

## References

[1] WHO. Cancer. 2018. Available from: https://www.who.int/news-room/fact-sheets/detail/cancer.

[2] Fallon MT, Colvin L. Neuropathic pain in cancer. Br J Anaesth 2013;111:105–111. 23794652 10.1093/bja/aet208

[3] Bennett MI, Rayment C, Hjermstad M, et al. Prevalence and aetiology of neuropathic pain in cancer patients: A systematic review. Pain 2012;153:359–365. 22115921 10.1016/j.pain.2011.10.028

[4] Niv D, Kreitler S. Pain and quality of life. Pain Pract 2001;1:150–161. 17129291 10.1046/j.1533-2500.2001.01016.x

[5] van den Beuken-van Everdingen MHJ, de Rijke JM, Kessels AG, et al. Prevalence of pain in patients with cancer: A systematic review of the past 40 years. Ann Oncol 2007;18:1437–1449. 17355955 10.1093/annonc/mdm056

[6] Brutman JN, Zhang S, Choi P, et al. Vapor cannabis exposure promotes genetic plasticity in the rat hypothalamus. Sci Rep 2019;9:1–12. 31728018 10.1038/s41598-019-53516-4 PMC6856070

[7] Buchwald D, Brønnum D, Melgaard D, et al. Living with a hope of survival is challenged by a lack of clinical evidence: An interview study among cancer patients using cannabis-based medicine. J Palliat Med 2020;23(8):1090–1093. 31710262 10.1089/jpm.2019.0298

[8] Davis MP. Cannabinoids for symptom management and cancer therapy: The evidence. JNCCN J Natl Compr Cancer Netw 2016;14:915–922. 10.6004/jnccn.2016.0094 27407130

[9] MacCallum CA, Russo EB. Practical considerations in medical cannabis administration and dosing. Eur J Intern Med 2018;49:12–19. 29307505 10.1016/j.ejim.2018.01.004

[10] Frieden TR. Evidence for health decision making-beyond randomized, controlled trials. N Engl J Med 2017;377:465–475. 28767357 10.1056/NEJMra1614394

[11] Honarmand K, Tierney MC, O'Connor P, et al. Effects of cannabis on cognitive function in patients with multiple sclerosis. Neurology 2011;76:1153–1160. 21444900 10.1212/WNL.0b013e318212ab0c PMC3068013

[12] Gruber SA, Sagar KA, Dahlgren MK, et al. Splendor in the grass? A pilot study assessing the impact of medical marijuana on executive function. Front Pharmacol 2016;7:355. 27790138 10.3389/fphar.2016.00355 PMC5062916

[13] Broyd SJ, Van Hell HH, Beale C, et al. Acute and chronic effects of cannabinoids on human cognition—A systematic review. Biol Psychiatry 2016;79:557–567. 26858214 10.1016/j.biopsych.2015.12.002

[14] Burggren AC, Shirazi A, Ginder N, et al. Cannabis effects on brain structure, function, and cognition: Considerations for medical uses of cannabis and its derivatives. Am J Drug Alcohol Abuse 2019;45:563–579. 31365275 10.1080/00952990.2019.1634086 PMC7027431

[15] Colizzi M, Bhattacharyya S. Does cannabis composition matter? Differential effects of delta 9-tetrahydrocannabinol and cannabidiol on human cognition. Curr Addict Reports 2017;4:62–74. 10.1007/s40429-017-0142-2 PMC5435777 28580227

[16] Curran VH, Brignell C, Fletcher S, et al. Cognitive and subjective dose-response effects of acute oral Δ9-tetrahydrocannabinol (THC) in infrequent cannabis users. Psychopharmacology (Berl) 2002;164:61–70. 12373420 10.1007/s00213-002-1169-0

[17] Liem-Moolenaar M, Beek ETT, Kam MLD, et al. Central nervous system effects of haloperidol on THC in healthy male volunteers. J Psychopharmacol 2010;24:1697–1708. 20142302 10.1177/0269881109358200

[18] Oomen PP, Van Hell HH, Bossong MG. The acute effects of cannabis on human executive function. Behav Pharmacol 2018;29:605–616. 30199388 10.1097/FBP.0000000000000426

[19] Volkow ND, Swanson JM, Evins AE, et al. Effects of cannabis use on human behavior, including cognition, motivation, and psychosis: A review. JAMA Psychiatry 2016;73:292–297. 26842658 10.1001/jamapsychiatry.2015.3278

[20] Janelsins MC, Kohli S, Mohile SG, et al. An update on cancer- and chemotherapy-related cognitive dysfunction: Current status. Semin Oncol 2011;38:431–438. 21600374 10.1053/j.seminoncol.2011.03.014 PMC3120018

[21] Janelsins MC, Kesler SR, Ahles TA, et al. Prevalence, mechanisms, and management of cancer-related cognitive impairment. Int Rev Psychiatry 2014;26:102–113. 24716504 10.3109/09540261.2013.864260 PMC4084673

[22] Pendergrass JC, Targum SD, Harrison JE. Cognitive impairment associated with cancer: A brief review. Innov Clin Neurosci 2018;15:36–44. 29497579 PMC5819720

[23] Adam KCS, Doss MK, Pabon E, et al. Δ-9-Tetrahydrocannabinol (THC) impairs visual working memory performance. Neuropsychopharmacology 2020;45:1807–1816. 32386395 10.1038/s41386-020-0690-3 PMC7608353

[24] Dahlberg K, Drew N, Nyström M. The Philosophy of Lifeworld Research. Reflective Lifeworld Research. Studentlitteratur: Lund; 2001.

[25] Stites SD, Harkins K, Rubright JD, et al. Relationships between cognitive complaints and quality of life in older adults with mild cognitive impairment, mild Alzheimer disease dementia, and normal cognition. Alzheimer Dis Assoc Disord 2018;32:276–283. 29944474 10.1097/WAD.0000000000000262 PMC6249095

[26] Kleckner AS, Kleckner IR, Kamen CS, et al. Opportunities for cannabis in supportive care in cancer. Ther Adv Med Oncol 2019;11:1–29. 10.1177/1758835919866362 PMC6676264 31413731

[27] Olla P, Rykulski N, Hurtubise JL, et al. Short-term effects of cannabis consumption on cognitive performance in medical cannabis patients. Appl Neuropsychol Adult 2021;28:647–657. 31790276 10.1080/23279095.2019.1681424

[28] Bar-Sela G, Zalman D, Semenysty V, et al. The effects of dosage-controlled cannabis capsules on cancer-related cachexia and anorexia syndrome in advanced cancer patients: Pilot Study. Integr Cancer Ther 2019;18:1534735419881498; doi: 10.1177/1534735419881498. 31595793 PMC6785913

[29] Huestis MA. Human cannabinoid pharmacokinetics. Chem Biodivers 2007;4:1770–1804. 17712819 10.1002/cbdv.200790152 PMC2689518

[30] Bruera E, Miller L, McCallion J, et al. Cognitive failure in patients with terminal cancer: A prospective study. J Pain Symptom Manage 1992;7:192–195. 1517640 10.1016/0885-3924(92)90074-r

[31] Moriarty O, McGuire BE, Finn DP. The effect of pain on cognitive function: A review of clinical and preclinical research. Prog Neurobiol 2011;93:385–404. 21216272 10.1016/j.pneurobio.2011.01.002

[32] Spindler M, Koch K, Borisov E, et al. The influence of chronic pain and cognitive function on spatial-numerical processing. Front Behav Neurosci 2018;12:1–10. 30123116 10.3389/fnbeh.2018.00165 PMC6085997

[33] Vytal KE, Cornwell BR, Letkiewicz AM, et al. The complex interaction between anxiety and cognition: Insight from spatial and verbal working memory. Front Hum Neurosci 2013;7:1–11. 23542914 10.3389/fnhum.2013.00093 PMC3610083

[34] Boychuk DG, Goddard G, Mauro G, et al. The effectiveness of cannabinoids in the management of chronic nonmalignant neuropathic pain: A systematic review. J Oral Facial Pain Headache 2015;29:7–14. 25635955 10.11607/ofph.1274

[35] Whiting PF, Wolff RF, Deshpande S, et al. Cannabinoids for medical use: A systematic review and meta-analysis. JAMA J Am Med Assoc 2015;313:2456–2473. 10.1001/jama.2015.6358 26103030

[36] Haroutounian S, Ratz Y, Ginosar Y, et al. The effect of medicinal cannabis on pain and quality-of-life outcomes in chronic pain: A prospective open-label study. Clin J Pain 2016;32:1036–1043. 26889611 10.1097/AJP.0000000000000364

[37] Wilkie G, Sakr B, Rizack T. Medical marijuana use in oncology. JAMA Oncol 2016;2:670–675. 26986677 10.1001/jamaoncol.2016.0155

[38] Abrams DI, Couey P, Shade SB, et al. Cannabinoid-opioid interaction in chronic pain. Clin Pharmacol Ther 2011;90:844–851. 22048225 10.1038/clpt.2011.188

[39] Colizzi M, McGuire P, Pertwee RG, et al. Effect of cannabis on glutamate signalling in the brain: A systematic review of human and animal evidence. Neurosci Biobehav Rev 2016;64:359–381. 26987641 10.1016/j.neubiorev.2016.03.010

[40] Mizrahi R, Watts JJ, Tseng KY. Mechanisms contributing to cognitive deficits in cannabis users. Neuropharmacology 2017;124:84–88. 28414051 10.1016/j.neuropharm.2017.04.018 PMC5540783

[41] Pope HG, Gruber AJ, Hudson JI, et al. Early-onset cannabis use and cognitive deficits: What is the nature of the association? Drug Alcohol Depend 2003;69:303–310. 12633916 10.1016/s0376-8716(02)00334-4

[42] Solowij N, Stephens RS, Roffman RA, et al. Cognitive functioning of long-term heavy cannabis users seeking treatment. J Am Med Assoc 2002;287:1123–1131. 10.1001/jama.287.9.1123 11879109

[43] Bar-Sela G, Tauber D, Mitnik I. Cannabis-related cognitive impairment: A prospective evaluation of possible influences on patients with cancer during chemotherapy treatment as a pilot study. Anticancer Drugs 2018;30:91–97. 10.1097/CAD.0000000000000685 30540595

[44] National Academies of Sciences, Engineering and Medicine. The Health Effects of Cannabis and Cannabinoids: Current State of Evidence and Recommendations for Research. National Academies of Sciences, Engineering and Medicine: Washington, DC, USA; 2017. 28182367

[45] Jacobus J, Tapert S. Brain morphological changes and early marijuana use. Pharm Des 2014;20:2186–2193. 10.2174/13816128113199990426 PMC3930618 23829363

[46] Giedd J, Blumenthal J, Jeffries NO, et al. Brain development during childhood and adolescence: A longitudinal MRI study. Nat Neurosci 1999;2:861–863. 10491603 10.1038/13158

[47] Wilson W, Mathew R, Turkington T, et al. Brain morphological changes and early marijuana use: A magnetic resonance and positron emission tomography study. J Addict Dis 2000;19:1–22. 10.1300/J069v19n01_01 10772599

[48] Ehrenreich H, Rinn T, Kunert HJ, et al. Specific attentional dysfunction in adults following early start of cannabis use. Psychopharmacology (Berl) 1999;142:295–301. 10208322 10.1007/s002130050892

[49] Stiglick A, Kalant H. Residual effects of chronic cannabis treatment on behavior in mature rats. Psychopharmacology (Berl) 1985;436–439. 3927340 10.1007/BF00429660

[50] Bilkei-Gorzo A, Albayram O, Draffehn A, et al. A chronic low dose of Δ9-tetrahydrocannabinol (THC) restores cognitive function in old mice. Nat Med 2017;23:782–787. 28481360 10.1038/nm.4311

[51] Sarne Y. THC for age-related cognitive decline? Aging (Albany NY) 2018;10:3628–3629. 30420585 10.18632/aging.101648 PMC6326663

[52] Sarne Y, Toledano R, Rachmany L, et al. Reversal of age-related cognitive impairments in mice by an extremely low dose of tetrahydrocannabinol. Neurobiol Aging 2018;61:177–186. 29107185 10.1016/j.neurobiolaging.2017.09.025

[53] Price DD, Finniss DG, Benedetti F. A comprehensive review of the placebo effect: Recent advances and current thought. Annu Rev Psychol 2008;59:565–590. 17550344 10.1146/annurev.psych.59.113006.095941

[54] Amanzio M, Benedetti F. Neuropharmacological dissection of placebo analgesia: Expectation activated opioid systems versus conditioning-activated specific subsystems. J Neurosci 1999;19:484–494. 9870976 10.1523/JNEUROSCI.19-01-00484.1999 PMC6782391

[55] Gupta U, Verma M. Placebo in clinical trials. Perspect Clin Res 2013;4:49. 23533982 10.4103/2229-3485.106383 PMC3601706

